# Prevalence of normal weight obesity and its cardiometabolic implications among government doctors in Gujarat, India: a cross-sectional study

**DOI:** 10.1186/s40842-024-00189-2

**Published:** 2024-09-25

**Authors:** Yogesh M, Nidhi Trivedi, Naresh Makwana, Pagadala Hari Priya PVM Krishna, Kadalarasu D

**Affiliations:** 1 Department of Community Medicine, Shri M P Shah Government Medical College, Jamnagar, Gujarat India; 2 Department of Community Medicine Jamnagar, Shri M P Shah Govt medical college, Jamnagar, Gujarat India; 3 Final year Student, Shri M P Shah Government Medical College, Jamnagar, Gujarat India

**Keywords:** Obesity, Normal weight obesity, Central obesity, Cardiometabolic risk, Doctors

## Abstract

**Background:**

Obesity is rising globally. Normal weight obesity (NWO) and normal weight central obesity (NWCO) despite normal BMI pose added metabolic risks. Limited data on these phenotypes among Indian doctors merits investigation. The present study aimed to assess the prevalence of overall obesity, NWO, NWCO, and their associations with cardiometabolic risks among doctors in Gujarat, India.

**Methods:**

It’s a Cross-sectional study among 490 doctors aged 20–60 years at a tertiary hospital. Anthropometry, blood pressure, fasting glucose, and lipids were assessed. NWO was defined as a BMI of 18.5–24.9 kg/m2 with a high body fat percentage. NWCO as normal BMI and increased waist circumference. Body composition was assessed using an Omron body composition analyzer.

**Results:**

The prevalence of overall obesity was 101 (20%), NWO 239 (48.7%), and NWCO 210 (42.8%). Mean BMI, blood pressure, glucose, and LDL increased from normal weight to NWO/NWCO groups (*p* < 0.05). NWO and NWCO had significantly higher odds of hypertension, dyslipidemia, and high fasting blood sugar compared to non-obese after adjusting for confounders.

**Conclusion:**

The high burden of overall obesity, NWO, and NWCO among doctors highlights the need for lifestyle interventions to mitigate long-term cardiometabolic disease risk.

## Introduction

Obesity has reached epidemic proportions globally, with over 650 million adults being obese. The burden is expected to grow in the coming years, especially in developing countries undergoing nutrition transition like India [1]. Obesity is an established risk factor for cardiometabolic diseases like diabetes, hypertension, dyslipidemia, and cardiovascular disease [2]. However, the relationship between obesity and cardiometabolic risk is complex. Increasing evidence indicates that not just overall obesity but also central/abdominal adiposity and normal-weight obesity contribute significantly to metabolic abnormalities [3].

Normal weight obesity (NWO), also called normal weight centrally obese, refers to individuals with normal body mass index (BMI 18.5–24.9 kg/m2) but excess body fat [4]. Using bioelectrical impedance, normal-weight obese persons are identified by high body fat percentage despite normal BMI [5]. Another subset is normal weight central obesity (NWCO) which is characterized by normal BMI but increased waist circumference indicating abdominal/visceral adiposity [6].

Increasing prevalence of NWO up to 30% has been reported in urban Asian populations [7, 8]. Limited research from India indicates 14–31% NWO and NWCO prevalence in Kerala and north India [9, 10]. NWCO is even more common affecting over half of normal-weight adults in some Asian studies [11]. Doctors as a professional group remain particularly understudied regarding obesity prevalence despite being role models for health promotion. Unhealthy lifestyles make them equally vulnerable to weight gain and metabolic complications [12].

Only three studies have assessed obesity prevalence among Indian doctors so far [13–15]. Obesity ranged from 31 to 48% using only BMI, thus likely underestimating true prevalence [16]. No study has evaluated NWO or NWCO among doctors in India. Globally as well, there is negligible focus on misclassified obesity phenotypes among healthcare professionals. Determining the prevalence of overall obesity along with NWO and NWCO will provide insights into the exact magnitude of the problem. Further, the association of these obesity phenotypes with cardiometabolic risk factors is inadequately understood in Indian populations. The current study aims to estimate the prevalence of overall obesity, NWO, and NWCO, and the association of these obesity phenotypes with cardiometabolic risk factors.

## Materials and methods

### Study design and setting

A cross-sectional study was conducted at a tertiary care teaching hospital in Gujarat on government doctors (Above 20 years).

### Sample size

Using a 95% confidence interval, 5% margin of error, and estimating 32% prevalence of obesity based on previous studies [10], the minimum sample size was calculated to be 348 using the formula n = z2pq/d2 (where z = 1.96 for 95% CI, *p* = 0.5, q = 1-p, and d = 0.05). Accounting for a 20% non-response rate, the final sample size was approximately 490.

### Sampling technique

Systematic random sampling was used to recruit government doctors aged 20–60 years working in various departments at the hospital. The sampling frame comprised a list of all eligible 700 doctors obtained from the hospital administration. The sampling interval was calculated as N/*n* = 700/490 ≈ 2. Every 2nd doctor on the list was approached for participation after selecting a random starting point.

### Eligibility criteria

Inclusion Criteria were government doctors aged above 20 years, working in various departments at the tertiary care hospital, and providing written informed consent. Exclusion Criteria were Pregnant women, known cases of any chronic disease like diabetes, hypertension, or heart disease, on prolonged medication for any illness, had any acute illness in the past 1 month, and declined consent.

### Study variables

#### Independent variables

1. BMI categories: Non-obese (BMI < 25 kg/m2), Obese (BMI ≥ 25 kg/m2), Normal weight obesity (BMI 18.5–24.9 kg/m2 with high body fat percent, BF% ≥ 20.6% in men, ≥ 33.4% in women), Normal weight central obesity (BMI 18.5–24.9 kg/m2 with high waist circumference, WC ≥ 90 cm in men, ≥ 80 cm in women).
2. Waist circumference: Measured in cm at the midpoint between the lower margin of the least palpable rib and iliac crest.

#### Dependent variables

1. Glycemic parameters: Fasting blood glucose, Glycated hemoglobin (HbA1c).
2. Lipid profile: Total cholesterol, Triglycerides, LDL cholesterol, HDL cholesterol.
3. Blood pressure: Systolic blood pressure, Diastolic blood pressure.

#### Confounding variables

1. Age: In years.
2. Gender: Male, Female.
3. Dietary habits: Vegetarian, Non-vegetarian.
4. Physical activity: Metabolic equivalent of task (MET) minutes/week.
5. Smoking status: Current smoker, Ex-smoker, Never smoked.
6. Alcohol intake: Drinks/week.

#### Operational definitions

1. Obesity: In this study, we used the Asia-Pacific BMI cut-offs to define obesity as BMI ≥ 25 kg/m², by the World Health Organization (WHO) expert consultation recommendations for Asian populations. This decision was based on substantial evidence that Asian populations have higher body fat percentages and greater abdominal obesity at lower BMIs compared to Western populations, putting them at higher cardiometabolic risk at BMIs considered normal or overweight by international standards. The WHO expert consultation in 2004 and subsequent Asia-Pacific guidelines proposed lower BMI cut-offs for overweight (23–24.9 kg/m²) and obesity (≥ 25 kg/m²) in Asian populations to account for this ethnic variation in body composition and disease risk [17].
2. Normal Weight Obesity: BMI 18.5–24.9 kg/m2 and BF% ≥ 20.6% in men, ≥ 33.4% in females [18].
3. Normal Weight Central Obesity: BMI 18.5–24.9 kg/m2 and waist circumference ≥ 90 cm in males, ≥ 80 cm in females [19].
4. Hypertension: Systolic BP ≥ 140 mmHg and/or diastolic BP ≥ 90 mmHg or on antihypertensive medication [20].
5. Prehypertension: Systolic BP 120–139 mmHg and/or diastolic BP 80–89 mmHg without medication [20].
6. Dyslipidemia: Serum triglycerides ≥ 150 mg/dL, total cholesterol ≥ 200 mg/dL, LDL cholesterol ≥ 100 mg/dL, HDL cholesterol < 40 mg/dL in males and < 50 mg/dL in females [21].
7. Impaired fasting glucose: Fasting plasma glucose 100–125 mg/dL [22].
8. Diabetes: Fasting plasma glucose ≥ 126 mg/dL or HbA1c ≥ 6.5% or on antidiabetic medication [22].

### Data collection

A pre-tested, structured questionnaire was used to collect socio-demographic and clinical details from the study participants after taking written informed consent. Information on potential confounding factors like diet, physical activity, smoking status, and alcohol use was also gathered through the questionnaire.

Anthropometric measurements including height, weight, and waist circumference were measured using standard protocols. Height was measured to the nearest 0.1 cm using a portable stadiometer with the participant standing upright without shoes. Weight was recorded to the nearest 0.1 kg using a digital weighing scale with minimal clothing and no footwear. Body mass index (BMI) was calculated as weight (kg) divided by height squared (m^2^).

Waist circumference (WC) was measured at the midpoint between the lower margin of the least palpable rib and the top of the iliac crest using stretch-resistant tape [23]. It was measured to the nearest 0.1 cm with the participant standing upright, feet close together, and arms relaxed at the sides. Two readings were taken and the average was considered.

Body composition was assessed using a bioelectrical impedance analyzer (Omron HBF-720, Omron Corporation, Kyoto, Japan) as per standard protocol [24]. Based on the inbuilt prediction equations, the device estimates body fat percentage along with other parameters. Participants were asked to avoid heavy exercise, food, and beverages for 4 h before the test. Readings were taken with the participant standing barefoot on the analyzer platform, legs and thighs not touching, and arms extended forward.

Blood pressure was recorded using a mercury sphygmomanometer with the appropriate cuff size after 5 min of rest in a sitting position. Two readings were measured at an interval of 3 min and the mean was considered. If the readings differed by more than 10 mmHg, a third reading was taken.

Fasting blood samples were collected by trained phlebotomists after 8–12 h overnight fast. About 5 ml of venous blood was drawn and evenly distributed into a plain vacutainer for serum lipid analysis and a fluoride vacutainer for plasma glucose estimation. Within 30 min, the samples were centrifuged at 2500–3000 rpm for 15 min. The serum and plasma were separated and stored at -20 °C until shipment to the central laboratory. All biochemical tests were done at an internationally accredited laboratory certified by the National Accreditation Board for Testing and Calibration Laboratories using autoanalyzers (Roche Diagnostics, Germany). Serum total cholesterol, triglycerides, and HDL cholesterol were measured enzymatically, while LDL cholesterol was calculated using the Friedewald formula [25]. Plasma glucose was estimated by the hexokinase method.

International Physical Activity Questionnaire (IPAQ): Evaluates physical activity levels over the last 7 days across leisure time, work, transportation, and household domains. Total metabolic equivalent (MET-min/week) scores classify PA as low, moderate, or high levels. The IPAQ has acceptable measurement properties (Spearman’s rho 0.8, criterion validity 0.3) for assessing activity levels in 18-65-year-old adults [26]. 

### Data analysis

Data was analyzed using Statistical Package for Social Sciences (SPSS) version 21. Descriptive statistics like mean and standard deviation for continuous variables, and frequency and percentage for categorical variables were used. The chi-square test was applied to find the association between categorical variables. Logistic regression was used for multivariate analysis to determine predictors of outcomes. Pearson correlation analyses were carried out. A *P*-value < 0.05 was considered statistically significant.

### Ethical considerations

The study was approved by Shri MP Shah Medical College and GG Hospital Ethical Committee vide letter no 195/05/2022 on 13/01/2023. Written informed consent was obtained for participation in the study and use of the patient data for research and educational purposes. The procedures in the study follow the guidelines laid down in the Declaration of Helsinki (2008).

## Results

Table 1 presents the socio-demographic characteristics of the study participants stratified by obesity phenotypes. The mean age progressively increased from 32 years in the non-obese group to 42 years in the obese group (*p* < 0.001). The majority (60%) of the participants were male, with a higher proportion of males in the obese (67%) and NWO + NWCO (64%) groups compared to non-obese (45%). Most participants (73%) were non-vegetarians, and this percentage was higher in the obese (80%) and NWO + NWCO (77%) groups.

As measured by MET minutes per week, physical activity levels showed a decreasing trend from 2800 in the non-obese group to 1400 in the obese group (*p* < 0.001). The prevalence of current smoking was higher in the obese group (28%) compared to the non-obese group (10%). Similarly, the mean alcohol intake (drinks per week) was highest in the obese group (3.1) and lowest in the non-obese group (1.2), with a significant difference across groups (*p* = 0.02).

**Table 1:** Socio-demographic characteristics of the study participants:

Characteristics	Non-obese (*n* = 49)	NWCO only (*n* = 101)	NWO only (*n* = 130)	NWO + NWCO (*n* = 109)	Obese (*n* = 101)	Total (*n* = 490)	*P*-value
**Age (years), mean (SD)**	32 (5.2)	35 (6.1)	38 (5.8)	40 (6.5)	42 (7.3)	38 (6.8)	< 0.001**
**Gender, n (%)**							0.002**
Male	22 (45%)	58 (57%)	75 (58%)	70 (64%)	68 (67%)	293 (60%)	
Female	27 (55%)	43 (43%)	55 (42%)	39 (36%)	33 (33%)	197 (40%)	
**Dietary habits, n (%)**							0.003**
Vegetarian	18 (37%)	32 (32%)	35 (27%)	25 (23%)	20 (20%)	130 (27%)	
Non-vegetarian	31 (63%)	69 (68%)	95 (73%)	84 (77%)	81 (80%)	360 (73%)	
**Physical activity (MET-min/week), mean (SD)**	2800 (1200)	2200 (950)	1900 (820)	1600 (720)	1400 (680)	1980 (920)	< 0.001**
**Smoking status, n (%)**							0.01*
Current smoker	5 (10%)	12 (12%)	18 (14%)	22 (20%)	28 (28%)	85 (17%)	
Ex-smoker	3 (6%)	8 (8%)	12 (9%)	15 (14%)	18 (18%)	56 (11%)	
Never smoked	41 (84%)	81 (80%)	100 (77%)	72 (66%)	55 (54%)	349 (71%)	
**Alcohol intake (drinks/week), mean (SD)**	1.2 (2.1)	1.8 (2.5)	2.2 (2.8)	2.6 (3.1)	3.1 (3.5)	2.2 (3.0)	0.02*

*P-value < 0.05 – significant

**P-value < 0.001 – highly significant, the “non-obese” group (which we now call “normal weight”) includes individuals with BMI 18.5–24.9 kg/m² who do not have NWO or NWCO.

Table 2 presents the bivariate analysis between cardiometabolic risk factors and obesity phenotypes. Regarding glycemic parameters, the mean fasting blood sugar (FBS) increased progressively from 92 mg/dL in the non-obese group to 115 mg/dL in the NWO + NWCO group (*p* = 0.001). The proportion of participants with high FBS (> 100 mg/dL) was highest in the NWO + NWCO group (87%) and lowest in the non-obese group (20%) (*p* < 0.001).

**Table 2:** Bivariate analysis between cardiometabolic risk factors and obesity phenotypes

Parameter	Non-obese (*n* = 49)	NWCO only (*n* = 101)	NWO only (*n* = 130)	NWO + NWCO (*n* = 109)	Obese (*n* = 101)	Total (*n* = 490)	*P*-value
Glycaemic Parameters							
Mean FBS (SD) mg/dl	92 (8.2)	102 (12.5)	112 (22.1)	115 (19.8)	108 (18.5)	107 (18.2)	0.001*
High FBS (> 100 mg/dl), n (%)	10 (20%)	45 (45%)	78 (60%)	95 (87%)	32 (32%)	260 (53%)	< 0.001**
FBS (90–100 mg/dl), n (%)	12 (24%)	22 (22%)	10 (8%)	8 (7%)	18 (18%)	70 (14%)	0.04*
FBS (< 90 mg/dl), n (%)	27 (55%)	34 (34%)	42 (32%)	6 (6%)	51 (50%)	160 (33%)	0.01*
Blood Pressure Parameters							
Mean SBP	122 (17)	125 (12)	129 (21)	133 (22)	126 (18)	128 (19)	0.026*
Mean DBP	77 (11)	80 (9)	81 (14)	84 (13)	79 (13)	80 (12)	0.03*
Hypertension, n (%)	5 (10%)	15 (15%)	35 (27%)	45 (41%)	20 (20%)	120 (24%)	< 0.001**
Prehypertension, n (%)	10 (20%)	25 (25%)	40 (31%)	35 (32%)	30 (30%)	140 (29%)	0.04*
No hypertension, n (%)	34 (69%)	61 (60%)	55 (42%)	29 (27%)	51 (50%)	230 (47%)	< 0.001**
Lipid Profile							
Mean TC (SD) mg/dl	173 (34)	178 (29)	183 (40)	189 (41)	180 (37)	181 (37)	0.044*
Mean LDL (SD) mg/dl	100 (32)	105 (30)	120 (38)	125 (40)	110 (35)	112 (36)	< 0.001**
Mean HDL (SD) mg/dl	50 (10)	45 (8)	42 (9)	40 (7)	44 (11)	44 (10)	< 0.001**
Dyslipidemia, n (%)	15 (31%)	35 (35%)	75 (58%)	70 (64%)	45 (45%)	240 (49%)	< 0.001**

*P-value < 0.05 – significant**P-value < 0.001 – highly significant, Abbreviations: NWCO, Normal Weight Central Obesity; NWO, Normal Weight Obesity; SD, Standard Deviation; FBS, Fasting Blood Sugar; SBP, Systolic Blood Pressure; DBP, Diastolic Blood Pressure; TC, Total Cholesterol; LDL, Low-Density Lipoprotein; HDL, High-Density Lipoprotein.

For blood pressure parameters, the mean systolic blood pressure (SBP) and diastolic blood pressure (DBP) were highest in the NWO + NWCO group (133 mmHg and 84 mmHg, respectively) compared to the non-obese group (122 mmHg and 77 mmHg, respectively) (*p* = 0.026 for SBP and *p* = 0.03 for DBP). The prevalence of hypertension was highest in the NWO + NWCO group (41%) and lowest in the non-obese group (10%) (*p* < 0.001).

Regarding the lipid profile, the mean total cholesterol (TC) and low-density lipoprotein cholesterol (LDL-C) levels were highest in the NWO + NWCO group (189 mg/dL and 125 mg/dL, respectively) and lowest in the non-obese group (173 mg/dL and 100 mg/dL, respectively) (*p* = 0.044 for TC and *p* < 0.001 for LDL-C). The mean high-density lipoprotein cholesterol (HDL-C) level was lowest in the NWO + NWCO group (40 mg/dL) and highest in the non-obese group (50 mg/dL) (*p* < 0.001). The prevalence of dyslipidemia was highest in the NWO + NWCO group (64%) and lowest in the non-obese group (31%) (*p* < 0.001).

Table 3 displays the association between conditions adjusted for age, gender, physical activity, diet, alcohol, and smoking. High FBS had 4.2 times higher odds in NWCO versus non-obese (*p* = 0.01). Hypertension and dyslipidemia had significantly higher odds in the NWO and NWO + NWCO groups compared to non-obese.

**Table 3:** Association between high fasting blood sugar (FBS), hypertension, and dyslipidaemia among different strata of obesity, *n* = 490

Dependent variables	NWCO only vs. Non-obese	NWO only vs. Non-obese	NWO + NWCO vs. Non-obese	Obese vs. Non-obese
Hypertension	1.6 (0.5–4.2)	3.4 (1.2–8.4) *	6.7 (2.3–15.6) **	2.3 (0.8–5.9)
Dyslipidemia	1.2 (0.6–2.4)	3.2 (1.5–6.4) *	4.3 (1.9–8.7) **	1.9 (0.9–3.8)
High FBS	4.2 (1.8–9.7) *	23.4 (8.2–41.6) **	31.2 (10.8–62.4) **	2.0 (0.9–4.3)

Values are presented as adjusted odds Ratio (AOR) (95% Confidence Interval). All analyses are adjusted for age, gender, physical activity, diet, alcohol, and smoking. *P-value < 0.05 – significant, **P-value < 0.001 - highly significant. Abbreviations: NWCO, Normal Weight Central Obesity; NWO, Normal Weight Obesity; SBP, Systolic Blood Pressure; DBP, Diastolic Blood Pressure; TG, Triglycerides; TC, Total Cholesterol; LDL, Low-Density Lipoprotein; HDL, High-Density Lipoprotein; FBS, Fasting Blood Sugar.

**Fig. 1:**
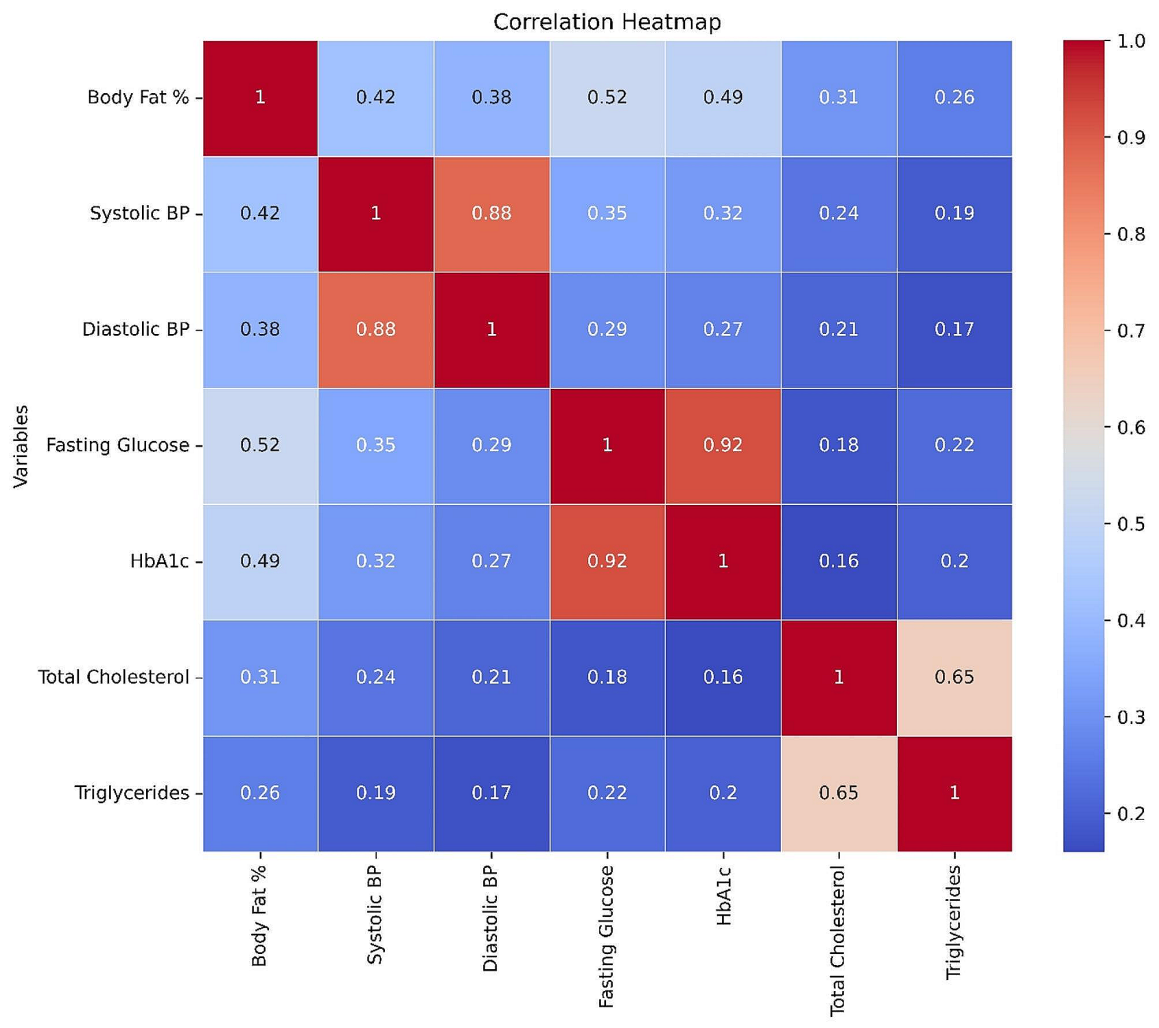
Correlation heatmap between body fat% and other cardio-metabolic risk factors

Fig. 1 illustrates the correlation heatmap between body fat percentage (BF%) and various cardiometabolic risk factors. The color gradient represents the strength and direction of the correlation, with darker shades indicating stronger positive correlations and lighter shades indicating weaker or negative correlations.

Body fat percentage has a moderate positive correlation with systolic BP (0.42), diastolic BP (0.38), fasting glucose (0.52), and HbA1c (0.49), suggesting that increased body fat is associated with higher blood pressure, glucose levels, and long-term blood sugar control.

Body fat percentage has a weaker positive correlation with total cholesterol (0.31) and triglycerides (0.26), indicating a less strong relationship with dyslipidemia.

Systolic BP and diastolic BP have a strong positive correlation (0.88), as expected.

Fasting glucose and HbA1c have a very strong positive correlation (0.92), as both are indicators of glycemic control.

Total cholesterol and triglycerides have a moderate positive correlation (0.65), as they are both lipid profile components.

On the other hand, BF% had a negative correlation with high-density lipoprotein cholesterol (HDL-C) (*r*=-0.28), suggesting that higher body fat percentage was associated with lower levels of HDL-C, which is considered a protective factor against cardiovascular disease.

The heatmap visually represents the relationships between body fat percentage and various cardiometabolic risk factors, highlighting the potential impact of excess body fat on metabolic health, even in individuals with a normal body mass index (BMI).

## Discussion

The present study found a high prevalence of overall obesity 101 (20%), NWO 239 (48.7%), and NWCO 210 (42.8%) among government doctors in Gujarat. The prevalence of obesity among doctors in our study was higher compared to 21% of obesity reported in Malaysia in health care workers [27]. However, it was lower than 48.4% overweight/obesity among medical students and doctors in Delhi [15]. The obesity prevalence among doctors mirrors the rising trend in the general Indian population from 11.8% in 1998 to 31.3% in 2016 based on National Family Health Surveys [28]. This highlights the influence of environmental factors like nutrition transition, sedentary lifestyles, and stress in predisposing doctors to obesity like the general population.

A key finding was the high prevalence of misclassified obesity phenotypes like NWO and NWCO among apparently normal-weight doctors. NWO prevalence was 37.4%, higher than 20.7% among Kerala adults [10] and 13.5% in north Indians [9]. NWCO prevalence was 46.2%, less than a previous s cross-sectional study in Shaanxi, China reported a NWCO prevalence of 58.3% [29]. This shows that nearly half the doctors with normal BMI had increased cardio-metabolic risk associated with excess abdominal fat. Reliance on BMI underestimates obesity and its risks as highlighted by the high burden of NWO and NWCO in our study.

Comparison of Obesity Prevalence Using Different BMI Cut-offs Our study, using Asia-Pacific BMI cut-offs, found a high prevalence of overall obesity (20%) among government doctors in Gujarat. However, to facilitate better comparison with global data, we also calculated obesity prevalence using the standard WHO cut-offs. When applying the international criteria (BMI ≥ 30 kg/m²), the prevalence of obesity in our sample drops to 7.8% (38 out of 490 doctors). This substantial difference (20% vs. 7.8%) underscores the impact of using region-specific versus standard BMI thresholds.

Similarly, our overweight category (BMI 23–24.9 kg/m²) based on Asia-Pacific guidelines would be reclassified as normal weight by international standards. This group, comprising 12.9% (63 out of 490) of our sample, would not be identified as at-risk using standard cut-offs, despite potentially having higher body fat and metabolic risks. Furthermore, our obese group (BMI ≥ 25 kg/m²) would be classified as overweight by WHO criteria, amounting to 20.6% (101 out of 490) of the doctors.

These discrepancies highlight the potential underestimation of obesity and its associated health risks in Asian populations when using standard BMI cut-offs. For instance, in the landmark study by Deurenberg-Yap et al., 46% of Singaporean Chinese had a BMI < 25 kg/m², yet their body fat percentages exceeded obesity thresholds [30]. Similarly, in our study, many doctors with normal BMI by international standards had high body fat (NWO) or central adiposity (NWCO), conditions linked to increased cardiometabolic risk.

We found NWO and NWCO were significantly associated with hypertension, dyslipidemia, and hyperglycemia compared to normal weight after adjusting for confounders. Another study showed higher odds of prediabetes and diabetes in NWO versus normal-weight individuals [31] and also a meta-analysis demonstrated a positive association of NWO with metabolic syndrome and its components [32]. Increased visceral adiposity in NWCO also predisposes to adverse cardiometabolic effects like insulin resistance and atherogenic lipid changes as suggested by previous research [33]. Our study provides novel data on NWO and NWCO associations with cardiometabolic risks in Indian doctors.

Mean SBP, DBP, blood glucose, and LDL cholesterol progressively increased from normal weight to NWO/NWCO groups in our study. Elevated BP among NWO individuals was seen in another Indian study [10]. Comparable trends of rising BP and metabolic derangements from normal weight to NWCO categories were observed among Japanese adults [11]. Our findings add to the limited evidence that normal-weight obesity phenotypes have similar or greater cardiometabolic abnormalities compared to conventional obesity.

Our findings contribute to the limited literature on obesity and its metabolic implications among healthcare professionals, particularly in the Indian context. While previous studies have primarily focused on BMI-based obesity estimates [14, 15], our comprehensive approach, including assessment of NWO and NWCO, provides novel insights into the often-overlooked aspects of misclassified obesity in this population.

The high burden of NWO and NWCO among doctors in our study highlights the need for regular assessment of body composition beyond BMI during health screenings. Incorporating more accurate techniques, such as DEXA scans, could further improve the identification of individuals at risk for obesity-related complications [5]. Furthermore, targeted interventions addressing the unique lifestyle challenges and occupational demands faced by healthcare professionals are warranted to promote weight management and mitigate cardiometabolic risks effectively [34, 35].

By addressing the obesity epidemic within the healthcare workforce, doctors can better serve as effective role models and catalysts for promoting healthier lifestyles and mitigating cardiometabolic disease risks in the broader community. Efforts to combat weight bias and stigma among medical professionals may also contribute to more effective obesity management strategies [36, 37].

Furthermore, nationwide screening programs and lifestyle interventions targeting cardiovascular and metabolic disorders, as suggested by Santulli et al. (2023), could yield significant public health benefits by identifying and addressing obesity-related risks at a population level, including among healthcare professionals [38]. 

### Limitations and strengths of the study

One significant limitation is selection bias. The study utilized sampling from a single tertiary care hospital, which may limit the generalizability of the findings to the broader population of doctors in Gujarat or India. The doctors at this particular hospital may have systematically different lifestyles, dietary habits, physical activity levels, or socioeconomic backgrounds compared to doctors working in other settings or regions.

Measurement bias is another concern. Although standardized techniques were employed for anthropometric measurements, blood pressure assessment, and biochemical analyses, the potential for measurement errors cannot be eliminated. Factors such as variations in technique, equipment calibration, or human error could lead to inaccuracies in the data collected. Specifically, the use of bioelectrical impedance for body composition analysis may have limitations in accurately estimating body fat percentage compared to more advanced techniques like dual-energy X-ray absorptiometry (DEXA) scans. Bioelectrical impedance equations can be influenced by factors such as hydration status, body build, and ethnicity, potentially introducing bias in the classification of normal-weight obesity (NWO).

Confounding is another limitation. While the analysis adjusted for major confounding variables like age, gender, dietary habits, physical activity levels, smoking, and alcohol consumption, there is a possibility of residual confounding from unmeasured or inadequately measured factors. Variables such as socioeconomic status, stress levels, sleep patterns, genetic predispositions, and other lifestyle factors could potentially influence both obesity phenotypes and cardiometabolic risk factors.

The cross-sectional nature of the study design limits the ability to establish causal relationships between obesity phenotypes and cardiometabolic risk factors. The temporal sequence of events cannot be determined, and reverse causality (where cardiometabolic abnormalities precede or contribute to the development of obesity phenotypes) cannot be ruled out. Additionally, information on dietary habits, physical activity levels, smoking status, and alcohol intake was obtained through self-reported questionnaires, which are susceptible to recall bias and social desirability bias. Participants may have inaccurately reported or underestimated their intake or behaviors, introducing measurement errors that could affect the observed associations.

Fourth, our reliance on a single fasting blood sample for metabolic assessments introduces potential misclassification bias. Day-to-day variations in glucose and lipid levels could lead to over- or underestimation of metabolic abnormalities.

Fifth, the high proportion of men in our sample (60%) could skew our findings if gender significantly modifies the relationship between obesity phenotypes and cardiometabolic risks.

Despite these limitations, the study has notable strengths. It is one of the first Indian studies to evaluate the prevalence of misclassified obesity phenotypes, such as normal-weight obesity (NWO) and normal-weight central obesity (NWCO), among doctors. It addresses a research gap by providing insights into the burden of these often-overlooked obesity phenotypes and their associations with cardiometabolic risk factors in a specific professional group.

The study comprehensively assessed various cardiometabolic risk factors, including blood pressure, fasting glucose, glycated hemoglobin (HbA1c), and a detailed lipid profile. This comprehensive approach allowed for a thorough evaluation of the associations between obesity phenotypes and multiple cardiometabolic parameters. Additionally, the study employed standardized protocols and techniques for anthropometric measurements, blood pressure assessment, and biochemical analyses, enhancing the reliability and comparability of the data collected.

Although bioelectrical impedance has limitations, its use for assessing body composition allowed for identifying individuals with normal-weight obesity, which would have been missed if only body mass index (BMI) was considered. This approach avoided underestimation of obesity prevalence compared to BMI-based studies. With a sample size of 490 doctors, the study had sufficient statistical power to detect meaningful differences in cardiometabolic risk factors across the various obesity phenotype groups.

The analyses adjusted for potential confounding variables, such as age, gender, dietary habits, physical activity levels, smoking, and alcohol consumption, aiming to account for the influence of these factors on the observed associations between obesity phenotypes and cardiometabolic risk. The findings of this study have important clinical and public health implications. By highlighting the high burden of overall obesity, NWO, and NWCO among doctors, the study underscores the need for lifestyle interventions and targeted strategies to mitigate long-term cardiometabolic disease risks in this specific population, who serve as role models for health promotion.

While the study has notable strengths, it is essential to acknowledge and discuss the potential limitations and biases transparently. This allows for a balanced interpretation of the findings and provides insights for future research to address these limitations and strengthen the evidence base further.

The high burden of overall obesity, normal weight obesity, and normal weight central obesity observed among doctors in the present study highlights the need to investigate the underlying factors contributing to these conditions within this profession. Doctors often face unique challenges that may predispose them to unhealthy weight gain and an increased risk of cardiometabolic complications. Long working hours, irregular schedules, and high stress can disrupt healthy eating patterns and physical activity routines. Furthermore, the passive nature of clinical work and limited opportunities for regular exercise may contribute to excess body fat and abdominal adiposity accumulation. Additionally, the easy availability of unhealthy food options within hospital settings and the normalization of poor dietary habits due to the demanding nature of the profession could exacerbate the problem. Addressing these occupational and lifestyle factors through targeted interventions, such as promoting stress management techniques, providing access to healthy food options, and encouraging regular physical activity, may be crucial in mitigating the rising burden of obesity and related cardiometabolic risks among doctors.

## Conclusion

The present study found a high prevalence of overall obesity, NWO, and NWCO among doctors in India. Despite being aware of health risks, obesity predisposes doctors to cardiovascular and metabolic complications like the general population. Only about one-third had normal weight, while the majority had conventional or misclassified obesity phenotypes. NWO and NWCO were significantly associated with adverse impacts on blood pressure, glycemic status, and lipid profile.

These findings underscore the urgent need for health promotion interventions like improving diet quality, increasing physical activity, and managing stress among doctors. Periodic assessment of body composition along with BMI is required during health screenings. As role models and disease prevention experts, maintaining healthy lifestyles and ideal weight should be priorities among doctors themselves. This will enable them to curb escalating obesity rates and related complications in the communities they serve.

## Data Availability

The datasets generated and/or analyzed during the current study are not publicly available to protect the privacy of the study participants but are available from the corresponding author upon reasonable request.
